# Association of lipid profile and reported edentulism in the elder population: data from the China Health and Retirement Longitudinal Study

**DOI:** 10.1186/s12903-022-02492-9

**Published:** 2022-10-15

**Authors:** Shuping Wang, Yutao Wang, Riyue Yu, Dingxiang Yuan, Yaofeng Ni, Lixin Wang, Man Sun, Xin Wang

**Affiliations:** 1 Department of Stomatology, Beijing Shijitan Hospital, Capital Medical University, Beijing, China; 2 Shanghai Fufan Information Technology Co., Ltd, No.323 Guoding Road, Yangpu District, Shanghai, 200433 China; 3 Community Health Service Center, Huangcun Town, Daxing District, Beijing, China; 4 Department of VIP Dental Service, Beijing Stomatological Hospital, Capital Medical University, Beijing, China

**Keywords:** Lipid profile, Reported edentulism, LDL cholesterol, Elder population

## Abstract

**Objectives:**

Relationship between lipid profile and periodontitis has been reported. However, the association between lipid parameters and edentulism is unclear. This study aimed to investigate the association between lipid profile and reported edentulism in the elder population using a national cohort.

**Methods:**

A total of 3 100 participants aged 65 or above were enrolled in 2011 from China Health and Retirement Longitudinal Study, which was a national population-based survey. We used adjusted logistics models to investigate the relationship between lipid profile and reported edentulism before and after propensity score matching.

**Results:**

The mean (SD) age was 71.96 (5.63) years, and 1 581 (51.0%) were men. There were 254 (8.2%) individuals reporting edentulism, and the low-density lipoprotein cholesterol (LDL-C) was significantly higher in the reported edentulism group, compared with the non-edentulism (122.48 vs. 116.91 mg/dl, P = 0.015). In the multivariable model, LDL-C was significantly associated with a higher odds of reported edentulism (adjusted OR [95% CI], 1.004 [1.001–1.008]). In the matched population, LDL-C, non high-density lipoprotein cholesterol, remnant cholesterol, total cholesterol and triglycerides were positively associated with reported edentulism, while HDL-C was negatively associated.

**Conclusions:**

Lipid profiles are probably associated with edentulism, indicating the interaction between oral health and metabolic status in the elder population.

## Introduction

Oral diseases are among the most prevalent diseases worldwide and have serious health, mental and economic burdens [1]. Dental caries and periodontal disease are two major oral diseases impacting the quality of life globally [2]. Edentulism and tooth loss are severe consequences of these diseases and occur throughout life, especially in the elder population. Although the epidemiology studies show a gradual decline in the prevalence and incidence of edentulism and tooth loss at the global, regional, and country levels [3], the prevalence still increases with aging showing an incidence peak at 65 years [4].

Edentulism and tooth loss are closely related with obesity [5], diabetes [6], dementia [7, 8], chronic obstructive pulmonary disease [9], nonalcoholic fatty liver disease [10] and cancer [11, 12]. Dyslipidaemias are alterations in the lipid profiles which are independently associated with cardiovascular diseases, particularly elevated plasma low-density lipoprotein cholesterol (LDL-C) [13]. The global burden of dyslipidaemias is gradually increasing, and LDL-C level has ranked the 8th leading risk factor of mortality in 2019, which ranked 15th in 1990 [14]. There is emerging evidence regarding the interaction between lipid metabolism and systemic, local inflammation and inflammation related diseases [15–17]. Edentulism and tooth loss, as polymicrobial chronic inflammatory diseases, reflect sustained local inflammatory milieu [18], which play a potential role in the lipid metabolism and lipid level. A recent study reported the association between oral health conditions and changes in lipid profile among people aged 40 years or above [19]. However, the association of edentulism and lipid levels remains unclear in the elder population with the highest risk of tooth loss and edentulism.

Triglycerides and cholesterol perform distinct functions in the body, and are important markers for health. Triglycerides are a most common type of fat or lipid in the blood storing excess energy. Cholesterol is a waxy substance embedded in the lipoprotein, generally classified into high-density lipoprotein cholesterol (HDL-C), LDL-C and remnant cholesterol according to the density. In this study, we aimed to investigate the association of reported edentulism with lipid parameters in the elder population using a national cohort.

## Materials and methods

### Data source

Participants of the China Health and Retirement Longitudinal Study (CHARLS) at baseline visit between were included in this study. CHARLS is a public open research cohort database collecting a wide range of social-economic data and personal health information (http://charls.pku.edu.cn/). CHARLS covers a nationally representative sample of Chinese residents ages 45 and older to promote research on the elderly. The baseline national wave of was initiated in 2011 and included about 10 000 households and 17 500 individuals in 150 counties/districts and 450 villages/resident committees using multi-stage stratified probability proportionate to size (PPS) sampling.

**Fig. 1:**
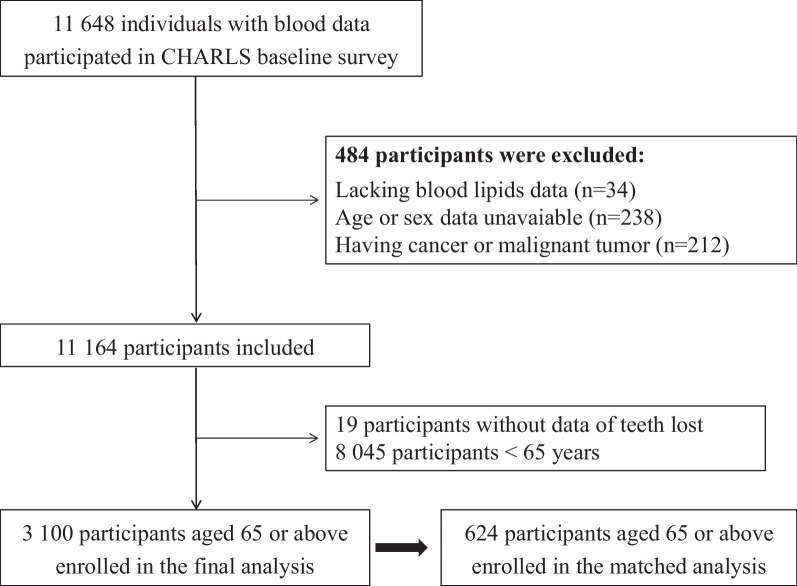
Flowchart of this current study

This study was in accordance with the principles of the Declaration of Helsinki and was approved by the Ethics Committee of Capital Medical University (grant number: 2020SY031). All participants provided their written informed consents before participating in the current study. The following criteria were required: (1) taking blood lipid examinations; (2) reporting oral health and tooth status; (3) without history of any malignancy; (4) aged 65 years or above. Figure 1 shows the design and flowchart of this current study. Finally, there were 3 100 individuals enrolled in the final analysis.

### Measurements and definitions

The demographic characteristics, lifestyles, and health status were acquired using the standard questionnaire, including age, sex, education level, marital status, smoking status, drinking status and self-reported health conditions. Educational level was categorized as ‘primary school or below’, ‘middle school’, and ‘high school or above’. Marital status included ‘married’, ‘unmarried’ and ‘others’. Smoking status was defined as ‘never smoking’, ‘current smoker’, and ‘former smoker’. Drinking status was defined as ‘current drinking more than once per month’, ‘current drinking once or less than once per month’, and ‘no current drinking’. Self-reported health conditions included tooth loss status and the diagnosis of hypertension, diabetes and dyslipidemia. Body mass index (BMI) was calculated as weight (kg)/[height (m)*height (m)]. The definition of obesity was BMI ≥ 28.0 kg/m^2^ for the Asian population [20]. Blood laboratory tests were carried out using fasting venous blood samples, and the lipid profiles included triglycerides, total cholesterol, HDL-C and LDL-C. The nonHDL-C was calculated as total cholesterol minus HDL-C. The remnant cholesterol was calculated using the Martin-Hopkins equation using the Stata.do-file (https://www.ldlcalculator.com/). The reported edentulism was assessed by the following question: “Have you lost all of your teeth? (Yes / no)”.

### Statistical analysis

The characteristics of the population were stratified by reported edentulism or not. The basic characteristics were presented as the mean (standard deviation, SD) for continuous variables and number (proportion) for categorical variables. The differences were compared by student’s t-test test for continuous variables and chi-square test for categorical variables. The distributions of lipid profiles were presented using box-plots.

The unadjusted and adjusted logistics regression models were used to analyze the association of the lipid parameters with reported edentulism. To control the potential confounding factors, age, sex, education level, marriage status, smoking status, drinking, BMI level, hypertension or not, dyslipidemia or not, diabetes or not were adjusted in multivariable model. In addition, we used propensity score method to match controls for individuals of reported edentulism. The matching ratio was 1:2, and all the potential confounding factors mentioned above were considered in the matching process. The association between lipid parameters and reported edentulism was further explored in the matched population.

All the analyses above were analyzed using R software (version 4.1.0). The differences were considered statistically significant at two-side P < 0.05.

## Results

### Population characteristics

**Fig. 2:**
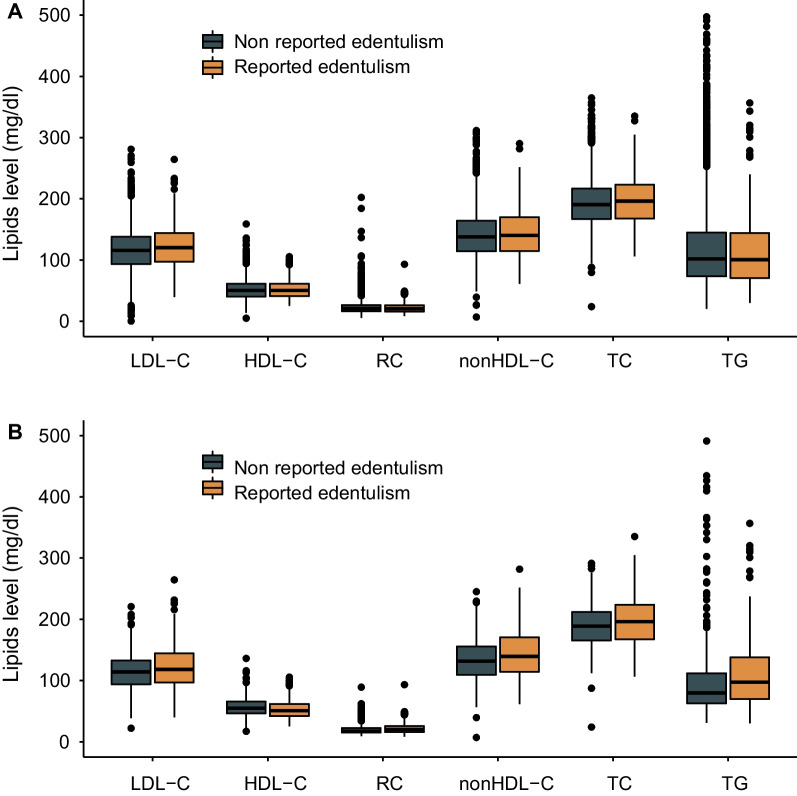
Distribution of lipid parameters in the whole and matched population

The mean (SD) age of the whole population was 71.96 (5.63) years, and 1 581 (51.0%) were men. There were 254 (8.2%) individuals with reported edentulism. The individuals of reported edentulism were older than people of non-edentulism (72.63 vs. 71.91, P = 0.049). There were no significant differences for sex, education, marriage, BMI, smoking, drinking and self-reported diagnosis of hypertension, diabetes and dyslipidemia as shown in Table 1. LDL-C was significantly higher in people with reported edentulism (122.48 vs. 116.91 mg/dl, P = 0.015), and no significant differences were observed for HDL-C, nonHDL-C, remnant cholesterol, total cholesterol and triglycerides (P > 0.05). In the matched population, 208 individuals with reported edentulism and 416 controls were selected, and the covariates were balanced as shown in Table 2. Of note, LDL-C (122.53 vs. 115.00 mg/dl, P = 0.009), nonHDL-C (145.14 vs. 134.02 mg/dl, P < 0.001), remnant cholesterol (22.40 vs. 20.16 mg/dl, P = 0.004), total cholesterol (197.76 vs. 190.47 mg/dl, P = 0.022) and triglycerides (116.30 vs. 101.64 mg/dl, P = 0.017) were significantly higher in the reported edentulism group, while HDL-C (52.62 vs. 56.45 mg/dl, P = 0.005) was significantly lower. The distributions of lipid profiles between the reported edentulism and non-edentulism groups in the whole population and matched population were shown in Fig. 2.

**Table 1:** Characteristics of this study

Characteristics	Non reported edentulism	Reported edentulism	P value
Participants, No	2846	254	
Age, years	71.91(5.58)	72.63(6.12)	0.049
Men, n (%)	1463(51.4)	118(46.5)	0.148
Education, n (%)			
Primary or below	2381(83.8)	223(88.1)	0.195
Middle school	282(9.9)	19(7.5)	
High school or above	177(6.2)	11(4.3)	
Marriage status, n (%)			
Married	2121(74.5)	181(71.3)	0.316
Unmarried	25(0.9)	1(0.4)	
Others	700(24.6)	72(28.3)	
Smoking status, n (%)			
Never	1618(56.9)	149(58.7)	0.831
Current	872(30.7)	76(29.9)	
Former	354(12.4)	29(11.4)	
Drinking status, n (%)			
> once a month	664(23.3)	53(20.9)	0.503
≤ once a month	173(6.1)	13(5.1)	
No drinking	2007(70.6)	188(74.0)	
Hypertension, n (%)	959(33.8)	77(30.3)	0.286
Dyslipidemia, n (%)	271(9.7)	18(7.2)	0.245
Diabetes, n (%)	182(6.4)	13(5.1)	0.491
BMI, kg/m2	23.42(3.99)	22.97(3.34)	0.106
Obesity, n (%)	270(11.1)	17(7.9)	0.181
Total cholesterol, mg/dL	193.30(38.34)	197.44(40.65)	0.101
LDL-C, mg/dL	116.91(34.52)	122.48(37.45)	0.015
HDL-C, mg/dL	52.02(15.91)	52.20(15.78)	0.865
nonHDL-C, mg/dL	141.27(38.15)	145.24(40.77)	0.115
Remnant cholesterol, mg/dL	23.34(11.62)	22.62(9.01)	0.338
Triglycerides, mg/dL	125.96(90.20)	118.57(70.62)	0.204

Data are presented as mean (SD), or number (proportion)

*BMI*, body mass index; *LDL-C*, low-density lipoprotein cholesterol; *HDL-C*, high-density lipoprotein cholesterol

**Table 2:** Characteristics of the matched population

Characteristics	Non reported edentulism	Reported edentulism	P value
Participants, No	416	208	
Age, years	72.08(5.59)	72.66(6.09)	0.235
Men, n (%)	165(39.7)	95(45.7)	0.177
Education, n (%)			
Primary or below	380(91.3)	183(88.0)	0.408
Middle school	24(5.8)	17(8.2)	
High school or above	12(2.9)	8(3.8)	
Marriage status, n (%)			
Married	290(69.7)	149(71.6)	0.751
Unmarried	4(1.0)	1(0.5)	
Others	122(29.3)	58(27.9)	
Smoking status, n (%)			
Never	256(61.5)	118(56.7)	0.51
Current	111(26.7)	63(30.3)	
Former	49(11.8)	27(13.0)	
Drinking status, n (%)			
> once a month	85(20.4)	45(21.6)	0.854
≤ once a month	19(4.6)	11(5.3)	
No drinking	312(75.0)	152(73.1)	
Hypertension, n (%)	120(28.8)	59(28.4)	0.975
Dyslipidemia, n (%)	19(4.6)	13(6.2)	0.48
Diabetes, n (%)	19(4.6)	12(5.8)	0.648
BMI, kg/m2	23.29(3.59)	23.04(3.34)	0.394
Obesity, n (%)	37(8.9)	17(8.2)	0.88
Total cholesterol, mg/dL	190.47(35.66)	197.76(40.93)	0.022
LDL cholesterol, mg/dL	115.00(31.45)	122.53(37.69)	0.009
HDL cholesterol, mg/dL	56.45(15.90)	52.62(16.06)	0.005
nonHDL cholesterol, mg/dL	134.02(34.90)	145.14(40.83)	< 0.001
Remnant cholesterol, mg/dL	20.16(8.86)	22.40(9.36)	0.004
Triglycerides, mg/dL	101.64(71.66)	116.30(72.38)	0.017

Data are presented as mean (SD), or number (proportion)

*BMI*, body mass index; *LDL-C*, low-density lipoprotein cholesterol; *HDL-C*, high-density lipoprotein cholesterol

### Association of lipid profiles and reported edentulism

**Fig. 3:**
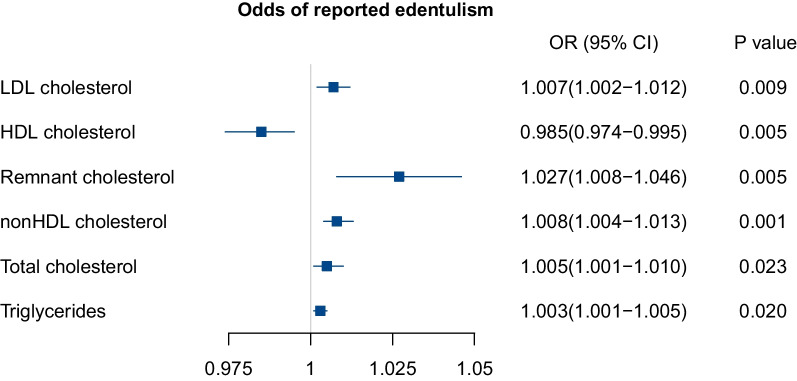
Association of lipid parameters and tooth loss risk in the matched population

In the whole population, LDL-C was significantly associated with a higher odds of reported edentulism, and the adjusted OR (95% CI) was 1.004 (1.001–1.008). We did not observe independent association between reported edentulism and other lipid parameters as shown in Table 3. In the matched population, LDL-C, HDL-C, nonHDL-C, remnant cholesterol, total cholesterol and triglycerides were all significantly associated with reported edentulism as shown in Fig. 3, and the adjusted OR (95% CI) values were 1.007 (1.002–1.012), 0.985 (0.974–0.995), 1.008 (1.004–1.013), 1.027 (1.008–1.046), 1.005 (1.001–1.010) and 1.003 (1.001–1.005) respectively.

**Table 3:** Association between lipid profiles and tooth loss in the whole population

	Unadjusted model		Adjusted model	
	OR (95% CI)	P value	OR (95% CI)	P value
LDL cholesterol	1.005(1.001–1.008)	0.014	1.004(1.001–1.008)	0.046
HDL cholesterol	1.001(0.992–1.008)	0.911	1.001(0.992–1.010)	0.838
Remnant cholesterol	0.994(0.981–1.006)	0.393	0.992(0.976–1.006)	0.302
nonHDL cholesterol	1.003(0.999–1.006)	0.099	1.002(0.999–1.006)	0.211
Total cholesterol	1.003(1.000–1.006)	0.092	1.003(0.999–1.006)	0.187
Triglycerides	0.999(0.997–1.001)	0.241	0.999(0.996–1.002)	0.172

*OR*, odds ratio; *CI*, confidence interval

Age, sex, education level, marriage status, smoking status, drinking, BMI level, hypertension or not, dyslipidemia or not, diabetes or not were adjusted in model

## Discussion

In this study, we found that reported edentulism was independently associated with lipid metabolism in the elder population, particularly LDL-C. In the matched population, six lipid parameters were significantly associated with reported edentulism including LDL-C, HDL-C, nonHDL-C, remnant cholesterol, total cholesterol and triglycerides.

The relationships between periodontitis and blood lipid parameters have been reported in different populations and regions. Previous studies showed that periodontitis was associated with low HDL-C, high triglycerides and total cholesterol [21–24]. On the contrary, some studies found no significant associations between periodontitis and lipid profiles [25, 26]. On the other hand, Song et al. found that periodontitis was both associated with baseline lipid profile and the longitudinal changes of blood lipid parameters [19]. The age levels of different populations could partially contribute to the inconsistent results. Kim et al. reported that the number of tooth loss was positively correlated with triglycerides level [27]. Our study provided further information on the association between lipid parameters and reported edentulism in the elder population.

The local and systematic inflammation may partially explain the underlying mechanism between edentulism and blood lipid parameters. Tooth loss causes the surrounding tissues damages and leads to chronic local inflammation [28–30]. Through the local tissue injury and chronic inflammation, oral bacteria could harm blood vessels or cause systemic inflammatory response [31–33]. In addition, control of the oral local infection is associated with a reduction in serum inflammatory markers including C-reactive protein (CRP) and interleukin-6 (IL-6) [34, 35]. These inflammatory reactions play an important role in the lipid metabolism [16], especially the atherogenic lipid components, such as LDL-C and remnant cholesterol [36, 37]. Similarly, the oral-derived systematic inflammatory is associated with glycemic control, complications, and incidence of diabetes, which have been reported in previous studies [38–40].

In our study, reported edentulism was positively associated with LDL-C, nonHDL-C, remnant cholesterol, total cholesterol and triglycerides, and negatively associated with HDL-C. Similarly, Meisel et al. [41] showed that statins use and reduction in LDL-C were associated with diminished tooth loss as a long-term response, which also supported the interaction effect between tooth loss or edentulism and lipid metabolism.

There were some limitations in this study. First, our study failed to collect the dietary information, as the dietary fat and cholesterol intake have an effect on plasma lipid profiles. Second, oral hygiene indicators, such as tooth brushing frequency were not considered in this current study, which could affect the local inflammatory response and lipid metabolism. Third, our study was a cross-sectional observational study, and we could not claim the causal association between edentulism and lipid parameters. Whether oral hygiene improvement could improve lipid level or lipid control could prevent edentulism remain to be investigated in further studies. Fourth, although there was a statistically significant association, the effect size was weak from the clinical perspective, indicating oral health as a systemic indicator of many health indicators. In the matched population, there were significant associations of LDL-C, HDL-C, nonHDL-C, remnant cholesterol, total cholesterol and triglycerides with reported edentulism. In the whole population, only LDL-C was significantly associated with reported edentulism probably due to the confounding bias. The effect size of lipid profile on edentulism needs further investigation in other studies.

## Conclusions

In conclusion, lipid profiles are probably associated with edentulism in the elder population. The interaction between oral condition and metabolic health needs further attention in clinical practice.

## Data Availability

The datasets used and/or analysed during the current study are available from the corresponding author on reasonable request.

## Abbreviations

CHARLS: China Health and Retirement Longitudinal Study
LDL-C: Low-density lipoprotein cholesterol
HDL-C: High-density lipoprotein cholesterol
BMI: Body mass index
SD: Standard deviation
CRP: C-reactive protein
IL-6: Interleukin-6
